# Inference of Locus-Specific Population Mixtures from Linked Genome-Wide Allele Frequencies

**DOI:** 10.1093/molbev/msae137

**Published:** 2024-07-03

**Authors:** Carlos S Reyna-Blanco, Madleina Caduff, Marco Galimberti, Christoph Leuenberger, Daniel Wegmann

**Affiliations:** Department of Biology, University of Fribourg, Fribourg 1700, Switzerland; Swiss Institute of Bioinformatics, Fribourg 1700, Switzerland; Department of Biology, University of Fribourg, Fribourg 1700, Switzerland; Swiss Institute of Bioinformatics, Fribourg 1700, Switzerland; Department of Biology, University of Fribourg, Fribourg 1700, Switzerland; Swiss Institute of Bioinformatics, Fribourg 1700, Switzerland; Department of Psychiatry, Yale University School of Medicine, New Haven, CT, USA; Veterans Affairs Connecticut Healthcare System, West Haven, CT, USA; Department of Mathematics, University of Fribourg, Fribourg 1700, Switzerland; Department of Biology, University of Fribourg, Fribourg 1700, Switzerland; Swiss Institute of Bioinformatics, Fribourg 1700, Switzerland

**Keywords:** gene flow, admixture, introgression rate, Gaussian process, linkage, hidden Markov model

## Abstract

Admixture between populations and species is common in nature. Since the influx of new genetic material might be either facilitated or hindered by selection, variation in mixture proportions along the genome is expected in organisms undergoing recombination. Various graph-based models have been developed to better understand these evolutionary dynamics of population splits and mixtures. However, current models assume a single mixture rate for the entire genome and do not explicitly account for linkage. Here, we introduce TreeSwirl, a novel method for inferring branch lengths and locus-specific mixture proportions by using genome-wide allele frequency data, assuming that the admixture graph is known or has been inferred. TreeSwirl builds upon TreeMix that uses Gaussian processes to estimate the presence of gene flow between diverged populations. However, in contrast to TreeMix, our model infers locus-specific mixture proportions employing a hidden Markov model that accounts for linkage. Through simulated data, we demonstrate that TreeSwirl can accurately estimate locus-specific mixture proportions and handle complex demographic scenarios. It also outperforms related *D*- and *f*-statistics in terms of accuracy and sensitivity to detect introgressed loci.

## Introduction

Gene flow, the exchange of genetic material between populations or different species (Slatkin 1985a), can occur through various mechanisms, such as migration, admixture, hybridization, cross-fertilization, or even by the dispersal of diaspores and pollinators (Barton and Hewitt 1985; Ellstrand et al. 2003; Tung and Barreiro 2017; Burgarella et al. 2019). This exchange may play a significant role in the maintenance of genetic variation, but also in the adaptation to multiple ecological niches (Anderson 1949; Slatkin 1985b, 1987; Rieseberg and Wendel 1993; Barton 2001). At sufficient levels, gene flow can lead to homogenization of populations, particularly in the face of opposing genetic drift (Ellstrand 2014). Gene flow might also increase genetic variation at a much higher rate than mutation (Grant and Grant 1994) and impact the process of speciation by becoming a primary source of genetic diversity and adaptive novelty for a population (Ellstrand et al. 2003; Abbott et al. 2013). Several genetic analyses have shown that gene flow, both ancient and present, is a common phenomenon in nature (Grant and Grant 1992; Mallet 2005; Patterson et al. 2006; Wegmann and Excoffier 2010; Tung and Barreiro 2017; Marchi et al. 2022), and a bifurcating tree, representing population or species historical relationships, fails to account for it (Kulathinal et al. 2009; Sousa et al. 2009; Green et al. 2010; Durand et al. 2011). This led to the development of methods that use allele frequency data and graph-based models to infer population splits and test for the presence of gene flow between divergent populations or species (Patterson et al. 2012; Pickrell and Pritchard 2012; Yang et al. 2012; Eaton and Ree 2013; Lipson et al. 2013; Martin et al. 2013; Lipson et al. 2014; Kozak et al. 2021), which, for instance, confidently settled the long-standing question whether gene flow occurred between modern humans and archaic hominins. However, these methods assume a genome-wide gene flow rate per migration edge, which is unrealistic in the presence of selection. In theory, the effective gene flow may vary significantly along the genome because of selection and genetic drift (Yamamichi and Innan 2012), making it essential to quantify these variations to better understand the dynamics that lead to introgression (Racimo et al. 2015, 2017; Suarez-Gonzalez et al. 2018; Sankararaman 2020).

Introgression is a lasting consequence of gene flow that leads to the assimilation of variants into the local gene pool through repeated back-crossing, resulting in their permanent inclusion (Anderson and Hubricht 1938). When introgressed loci increase the fitness of the recipient population, this is known as “adaptive introgression”. Unlike neutral introgression, which can be lost over time due to drift, adaptive introgression is sustained by selection and can eventually lead to fixation (Zhang et al. 2021). But selection may also prevent introgression if it reduces fitness (Christe et al. 2016). While introgressed loci may be identified through explicit demographic modeling (Luqman et al. 2021), the classic way is via population genetic summary statistics. Patterson’s *D*, for example, has been estimated in sliding windows along the genome to identify introgressed loci (Dasmahapatra et al. 2012; Kronforst et al. 2013; Smith and Kronforst 2013; Rheindt et al. 2014; Fontaine et al. 2015). Since it was originally intended for genome-wide analysis (Martin et al. 2015), more suitable related statistics have been used for analyzing specific short genomic regions, such as $f_{d}$ , $f_{dM}$, and $d_{\;f}$ (Malinsky et al. 2015; Martin et al. 2015; Pfeifer and Kapan 2019; Malinsky et al. 2021). There are other statistics, for instance, S* and its variants that use linkage disequilibrium information to detect long introgressed haplotypes (Plagnol and Wall 2006; Wall et al. 2009; Vernot and Akey 2014; Vernot et al. 2016; Browning et al. 2018) or ArchIE that combines diverse summary statistics to detect introgressed haplotypes without a reference (Durvasula and Sankararaman 2019, 2020). However, outlier scans based on such statistics are likely to ignore valuable information present in the full data, do not model linkage explicitly or require an arbitrary choice of large window size and outliers identification. To overcome these constraints, probabilistic frameworks such as hidden Markov models (HMMs) (Rabiner and Juang 1986; Prüfer et al. 2014; Seguin-Orlando et al. 2014; Skov et al. 2018; Steinrücken et al. 2018), and conditional random fields (CRFs) (Sankararaman et al. 2014) have been applied to infer the ancestry state of each site. These methods are extensions of models that infer local ancestry from genotyping data (Tang et al. 2006; Price et al. 2009; Wegmann et al. 2011; Lawson et al. 2012; Maples et al. 2013) and while explicitly accounting for demographic history and linkage, they rely on phased and training sequence data, unadmixed or archaic reference, and detailed demographic models. As a consequence, such approaches are not easily applicable to nonmodel species for which only limited data and knowledge is available.

To complement these methods, we here propose a model that makes use of Gaussian processes to infer locus-specific mixture proportions. Gaussian processes have a rather long history to model allele frequency differences between populations (Cavalli-Sforza and Edwards 1967; Felsenstein 1981), but have recently seen a surge in applications due to the development of the popular tool TreeMix (Pickrell and Pritchard 2012). Our method, TreeSwirl, explicitly takes an admixture graph (e.g. inferred by TreeMix) and genome-wide allele frequencies to infer locus-specific mixture proportions. To account for linkage, we make use of a HMM, wherein the hidden states are represented by the proportion of the mixture at a particular site and the observed data are represented by the sampled allele frequencies. To evaluate the performance of our method against other tools, we simulated data using various demographic models. We estimated the mixture proportions with TreeSwirl and computed related *D*- and *f*-statistics using *D*-suite Dinvestigate (Malinsky et al. 2021). Our findings revealed that TreeSwirl surpasses the summary statistics estimates in detecting the simulated signal of introgression under different scenarios, although at an additional computational cost. Furthermore, by applying TreeSwirl to real data cases, we successfully identified candidate genomic regions where migration rates fluctuate and may be subject to selection.

## Materials and Methods

### The Model

**Fig. 1.**
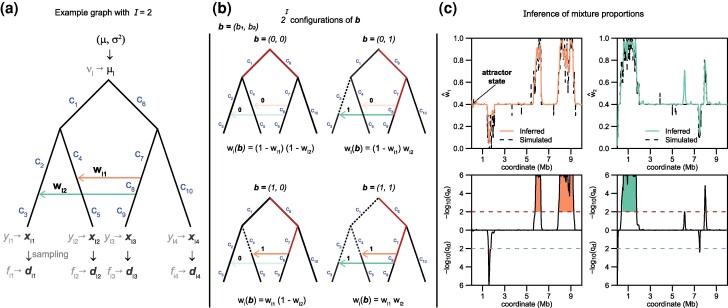
Inference example. a) Admixture graph with two migration edges marked in different colors. Parameters of interest are shown on the graph (root prior and branch lengths) as well as the untransformed and transformed ancient, sampling and population allele frequency variables. b) All possible configurations of b for two migration events when they are open or closed. c) Example of inference under our TreeSwirl model for each migration event. The top panel shows the posterior mean mixture proportions ${\hat{w}}_{l}$ compared to simulated estimates and the bottom panel shows the identified candidate regions under possible selection, where the false discovery rates for excess ($q_{e}$) and dearth ($q_{d}$) introgression was determined for each locus as explained in the “Inference” section.

Consider a set of populations m=1,2,…,M that are linked by a graph G which represents their population history in terms of population splits and migration events. Consider as well a series of diploid, biallelic loci l=1,2,…,L, where the total number of loci *L* might constitute, for instance, consecutive SNPs (single nucleotide polymorphisms) along the genome. At each locus *l*, a total number of $N_{l}=(N_{l1},\ldots ,N_{lM})$ alleles have been observed across the *M* populations, of which $n_{l}=(n_{l1},\ldots ,n_{lM})$ were derived and the remaining ancestral (or otherwise polarized). To model sampled allele counts $n_{l}|N_{l}$ we distinguish two processes: the first models the distribution of the vector of the actual but unknown population frequencies $y_{l}=(y_{l1},\ldots ,y_{lM}{)}^{\prime }$ given the graph G, and the second the distribution of the sampled allele counts $n_{l}|N_{l}$ given $y_{l}$ (Fig. 1a).

#### Evolution along the Graph G

We assume, as in Pickrell and Pritchard (2012), that the change in allele frequencies from the root to the tips of G is modeled as a Brownian motion (BM) process. For each locus *l*, the BM process starts at the root of G at a value of allele frequency which we denote by $\nu_{l}$. It proceeds along the branches of G and finally gives rise to the above-mentioned random vector $y_{l}$ at the leaves of G. The probability of $y_{l}$ is given by the multivariate normal density


$$\pi (y_{l}|\nu_{l},G)=N\left(\nu_{l},V(\nu_{l})\right),$$

where $\nu_{l}=(\nu_{l},\ldots ,\nu_{l}{)}^{\prime }$ is the mean vector and $V(\nu_{l})$ is the variance–covariance matrix corresponding to the BM on G. For the construction of $V(\nu_{l})$, which depends on the topology of G, the lengths of the branches in G and the migration rates, we follow Pickrell and Pritchard (2012). We set


(1) $$V(\nu_{l})=\nu_{l}(1-\nu_{l})W_{l},$$

where $W_{l}$ only depends on the graph topology, the branch lengths and the migration rates.

However, it was long recognized that BM with constant variance is not adequately describing allele frequency changes, especially close to boundaries and various transformations to alleviate the problem have been proposed (Felsenstein 1981). Here, we will consider the transformation


(2) $$\mu_{l}=\text{arcsin}(2\nu_{l}-1)$$

from the interval [0,1] onto [−π/2,π/2]. This has the advantage that all factors of $\nu_{l}(1-\nu_{l})$ in front of the variance matrices will be canceled. We thus replace (equation (1)) by


(3) $$W_{l}={\left(\frac{d\mu_{l}}{d\nu_{l}}\right)}^{2}V(\nu_{l}).$$

Let $x_{l}=(x_{l1},\ldots ,x_{lM}),x_{lm}=\text{arcsin}(2y_{lm}-1)$ denote the transformed population allele frequencies. The distribution of $x_{l}$ thus follows the multivariate normal density


(4) $$\pi (x_{l}|\mu_{l},G)=N\left(\mu_{l},W_{l}\right)$$

with $\mu_{l}=(\mu_{l},\ldots ,\mu_{l})=\mu_{l}1$.

The matrix $W_{l}$ is constructed as follows. Let G be a rooted population graph with *K* oriented branches k=1,…,K of length $c_{k}$, $c=(c_{1},\ldots ,c_{K}{)}^{\prime }$; the orientation of the branches points in direction of the leaves. We assume that the graph also contains *I* oriented migration edges $\tau_{i}$, i=1,…,I, to which we assign no branch length. The migration edges should be placed such that there are no cycles in the graph. However, we allow for bidirectional migration edges, which, to avoid cycles, we model effectively as two migration edges, of which the starting point of one precedes the end point of the other by an infinitesimally small branch.

We now consider paths leading from the root of the graph to a leaf taking some of the migration edges (open edges) and leaving others out (closed edges). More precisely, let


$$b=(b_{1},\ldots ,b_{I})$$

be a binary vector indicating a certain configuration of open and closed migration edges: a bit $b_{i}=1$ indicates that the migration edge $\tau_{i}$ is open and $b_{i}=0$ that the migration edge $\tau_{i}$ is closed (Fig. 1b). We denote by $w_{li}$ the migration rate, i.e. the probability of edge $\tau_{i}$ to be open, and thus we assign to the configuration b the probability


(5) $$w_{l}(b)=\prod_{i=1}^{I}w_{li}^{b_{i}}(1-w_{li}{)}^{1-b_{i}}.$$

Now, for a given configuration b, pick a population (leaf) *m* and a branch *k*. There is at most one path leading from the root to the population *m* and taking exactly the open migration edges according to b. If, moreover, this path contains the branch *k*, we set the indicator function $I_{mk}(b)$ equal to 1. Otherwise we set $I_{mk}(b)=0$.

Using this notation, we can now define the M×M-matrices $J_{lk}$ for each branch *k* element-wise by


(6) $${\left[J_{lk}\right]}_{mn}=\sum_{b}w_{l}(b)I_{mk}(b)\sum_{b^{\prime }}w_{l}(b^{\prime })I_{nk}(b^{\prime }),$$

where each sum runs over all the $2^{I}$ possible configurations of b and $b^{\prime }$, respectively. Each matrix $J_{lk}$ thus reflects the probabilities that branch *k* was common for any pair of leaves.

The matrix $W_{l}$, after all, is given by


(7) $$W_{l}(w)=\sum_{k=1}^{K}c_{k}J_{lk}.$$

This construction of the variance matrix $W_{l}(w_{l})$ is a generalized reformulation of an argument given in Pickrell and Pritchard (2012).

To unclutter the notation, we will use $W_{l}=W_{l}(w_{l})$ in the rest of this article and thus not indicate its dependence on the migration rates $w_{l}=(w_{l1},\ldots ,w_{lI})$.

#### Sampling

We assume that the observed allele counts $n_{lm}$ at locus *l* and population *m* follow a binomial distribution with parameters $N_{lm}$ and $y_{lm}$, where $y_{lm}$ is the true allele frequency in population *m*. By independence of the samples, we have


(8) $$\pi (n_{l}|y_{l})=\prod_{m=1}^{M}\text{Bin}(n_{lm}|N_{lm},y_{lm}).$$

If the sample sizes are sufficiently large, we can approximate this distribution by a multivariate normal density. Let $f_{l}=(f_{l1},\ldots ,f_{lM})$ with $f_{lm}=n_{lm}/N_{lm}$ denote the observed allele frequencies at locus *l*, which are approximately normally distributed with mean $y_{l}$ and a diagonal variance–covariance matrix:


(9) $$\text{diag}\left[\frac{y_{l1}(1-y_{l1})}{N_{l1}},\ldots ,\frac{y_{lM}(1-y_{lM})}{N_{lM}}\right].$$

The transformed observed allele frequencies $d_{l}=(d_{l1},\ldots ,d_{lM})$ with $d_{lm}=\text{arcsin}(2f_{lm}-1),$ are then approximated by the multivariate normal density


(10) $$\pi (d_{l}|x_{l})\approx N(x_{l},\Sigma_{l})$$

with


$$\Sigma_{l}=\text{diag}\left[\frac{1}{N_{l1}},\ldots ,\frac{1}{N_{lM}}\right]$$

because the factors $y_{l1}(1-y_{l1})$ are transformed away from the variance–covariance matrix (equation (9)) similar to equation (3).

#### Full Likelihood for One Locus

Given the ancestral frequency $\mu_{l}$, we obtain the likelihood by combining equation (4) and equation (10) and integrating out:


(11) $$\pi (d_{l}|\mu_{l},G)=\int \pi (d_{l}|x_{l})\pi (x_{l}|\mu_{l},G)dx_{l}.$$

Using well-known formulae for linear systems (see Theorem 4.4.1 in Murphy 2012), we obtain for the likelihood (equation (11)) the following approximation:


(12) $$\pi (d_{l}|\mu_{l},G)\approx N(\mu_{l},\Sigma_{l}+W_{l}).$$

We now set a normal prior on $\mu_{l}$, namely we assume that


$$\pi (\mu_{l})=N(\mu ,\sigma^{2}).$$

Again from Theorem 4.4.1 in Murphy (2012) we conclude that


(13) $$\pi (d_{l}|\mu_{l},\sigma^{2},G)=N(\mu_{l},S_{l})$$

with


(14) $$\mu_{l}=\mu_{l}1,\;S_{l}=\Sigma_{l}+W_{l}+\sigma^{2}11^{\prime }.$$

Explicitly


(15) $$\pi (d_{l}|\mu_{l},\sigma^{2},G)\approx \frac{1}{\sqrt{\left(2\pi {)}^{M}|S_{l}\right|}}\text{exp}\left[-\frac{\left(d_{l}-\mu_{l}1{)}^{\prime }S_{l}^{-1}(d_{l}-\mu_{l}1\right)}{2}\right].$$

We note that the above model accounts for variation in sample size among loci, but assumes that at least one sample was observed per locus and population.

### Hidden Markov Model

We develop a HMM for multiple loci l=1,…,L with varying migration rates for each of the *I* migration edges of graph G. We assume that the locus and specific migration rates $w_{li}$ take values out of a small set of discrete numbers between 0 and 1:


$$w_{li}\in \{w_{i1},w_{i2},\ldots ,w_{iJ_{i}}\}.$$

We thus have $J_{1}\cdot J_{2}\cdot \ldots \cdot J_{I}$ possible combinations and these combinations will constitute the hidden states of our Markov model. We denote the hidden state at locus *l* by $z_{l}$. Each state $z_{l}$ corresponds to a multiindex


$$j=(j_{1},j_{2},\ldots ,j_{I})$$

that defines the migration values $\left(w_{1j_{1}},\ldots ,w_{Ij_{I}}\right)$ of the migration edges. Thus, knowing the state $z_{l}$ is tantamount to knowing the combination of migration rates at the given site which in turn determines the matrix W in equation (7) via equation (5) and equation (6).

To account for linkage between loci, we assume that the locus-specific transition matrix $P(z_{l}=j^{\prime }|z_{l-1}=j)$ is based on physical or genetic distances $\delta_{l}$ between loci. We assume independence of the transition probabilities of the different migration edges:


$$P(z_{l}=j^{\prime }|z_{l-1}=j)=P_{l}(j,j^{\prime })=\prod_{i=1}^{I}P_{li}(j_{i},j_{i}^{\prime }).$$

Each one of the factors in this product is an element of a ladder-type Markov matrix $P_{li}$ which is defined via a transition rate matrix $\kappa_{i}\Lambda_{i}$:


(16) $$P_{li}=e^{\delta_{l}\kappa_{i}\Lambda_{i}}.$$

Here, $\kappa_{i}$ is a positive scaling parameter pertaining to migration edge *i*, the distances $\delta_{l}$ are known constants corresponding to the linkage distances. Further, the $J_{i}\times J_{i}$-matrices $\Lambda_{i}$ reflect a transition model for infinitesimal steps.

We consider two transition models: The first is a standard ladder-type model in which transitions are only allowed to neighboring states and at equal rates:


(17) $$\Lambda_{i}=\left(\begin{array}{cccccccc} -1 & 1 & 0 & 0 & \ldots & 0 & 0 & 0\\ 1 & -2 & 1 & 0 & \ldots & 0 & 0 & 0\\ 0 & 1 & -2 & 1 & \ldots & 0 & 0 & 0\\ \vdots & \vdots & \vdots & \vdots & & \vdots & \vdots & \vdots \\ 0 & 0 & 0 & 0 & \ldots & 0 & 1 & -1 \end{array}\right).$$

Under this transition matrix, only $\kappa_{i}$ is inferred, for which we maximize the Q-function using a linear search.

The second is also a ladder-type model, but which includes an attractor state $a_{i}\in \{w_{i1},\ldots ,w_{iJ_{i}}\}$ reflecting the background migration rate. Similar to Galimberti et al. (2020), we use two parameters to describe the fraction of loci deviating from the attractor state and the degree of that deviation, but choose a slightly different parametrization. Specifically, and given the two parameters $\phi_{i}$ and $\zeta_{i}$, we have


(18) $$\Lambda_{i}=\left(\begin{array}{ccccccccc} -1 & 1 & 0 & 0 & \ldots & 0 & 0 & 0 & 0\\ 1-\zeta_{i} & -2 & 1+\zeta_{i} & 0 & \ldots & 0 & 0 & 0 & 0\\ 0 & 1-\zeta_{i} & -2 & 1+\zeta_{i} & \ldots & 0 & 0 & 0 & 0\\ \vdots & \vdots & \vdots & \vdots & \ddots & \vdots & \vdots & \vdots & \vdots \\ 0 & 0 & 0 & 0 & \ldots & 1+\zeta_{1} & -2 & 1-\zeta_{i} & 0\\ 0 & 0 & 0 & 0 & \ldots & 0 & 1+\zeta_{i} & -2 & 1-\zeta_{i}\\ 0 & 0 & 0 & 0 & \ldots & 0 & 0 & 1 & -1 \end{array}\right)$$

with the attractor row given by


$$(\begin{array}{ccccccccc} 0 & \ldots & 0 & \phi_{i} & -2\phi_{i} & \phi_{i} & 0 & \ldots & 0). \end{array}$$

Note that the $\kappa_{i}$, $\phi_{i}$, and $\zeta_{i}$ all must be strictly positive. However, we limit $\phi_{i}$ and $\zeta_{i}$ to the range (0,1] to ensure that the stationary probability of the attractor state $a_{i}$ is higher than for any other state.

Finally, the emission probabilities are generated via the marginal likelihood (equation (15)):


(19) $$P(d_{l}|z_{l}=j)=\pi (d_{l}|\mu_{l},\sigma^{2},G_{\;j}),$$

where $G_{\;j}$ denotes the population graph with migration rates according to the state $z_{l}=j$ and $\mu_{l}$ is the root state at site *l*.

### Inference

We developed an empirical Bayes inference scheme for the hidden states under the assumption that the topology of the admixture graph is either known or was previously obtained. Specifically, we first infer both the emission and transition probabilities using the Baum–Welch algorithm (Baum et al. 1970) and then posterior state probabilities under the inferred parameters. We implemented the SQUAREM inference scheme (Varadhan and Roland 2008) for accelerated convergence of the Baum–Welch algorithm. As detailed in the Supplementary Information (see section “Inference”), the Baum–Welch algorithm requires numerical optimization in each iteration. While the parameter of the root prior *μ* can be optimized analytically, we resort to Newton–Raphson optimization (Nocedal and Wright 2006; Lange 2010) for the root prior $\sigma^{2}$ and for parameters of the population graph (i.e. the branch lengths c) and to Nelder–Mead optimization (Nelder and Mead 1965) for the parameters regarding the transition matrices with attractors (i.e. the $\kappa_{i}$, $\phi_{i}$, $\zeta_{i}$) or a linear search for transition matrices with no attractors (i.e. the $\kappa_{i}$).

The Baum–Welch algorithm may be sensitive to initial conditions. We obtain initial estimates of all parameter values as follows (see Supplementary Information for more details):

1. We use the observed variance–covariance matrix of the transformed observed frequencies as an initial guess of the variance–covariance matrix W.
2. To account for variation in W among loci, we refine this initial estimate using a Gaussian mixture model (GMM) under which the transformed observed frequencies are modeled by one of r=1,…,R multivariate Gaussian distributions with variance–covariance matrices $W_{r}$ but shared root priors *μ* and $\sigma^{2}$. This model assumes no constraints regarding the structure of the $W_{r}$ and can be optimized with an expectation–maximization (EM) algorithm with analytic updates. The hidden states $s=(s_{1},\ldots ,s_{L})$ with $s_{l}\in \{1,\ldots ,R\}$ are assumed to follow a Categorical distribution s∼Cat(π) with $\pi =(\pi_{1},\ldots ,\pi_{R})$.
3. We next use a Nelder–Mead algorithm to coerce the inferred variance–covariance matrices $W_{1},\ldots ,W_{R}$ onto the population graph. Specifically, we seek to find the set of branch lengths c and partition-specific migration rate $w_{r}$ that best explain the previously learned variance–covariance matrices using the weighted residuals sum of squares (RSS). To mitigate convergence problems, we repeat the Nelder–Mead *C* times and use different initial values each time. We set C=1,000 by default.
4. To determine the optimal number of mixture components *R*, we repeat steps 2 and 3 with an increasing number of mixture components R=1,2,3,4,… as long as (i) each $\pi_{r}$ is larger than some threshold, which we set to 0.2 by default, and (ii) the weighted RSS is lower than the weighted RSS of R−1.
5. We then run a Baum–Welch algorithm to infer the branch lengths c, the root prior *μ* and $\sigma^{2}$ and the transition parameters $\kappa_{i}$ for the attractor-free, ladder-type transition matrix (equation (17)). Upon convergence, we identify the top *T* states with the highest average posterior probabilities across all loci, and consider each of these states as a potential attractor. We set T=5 by default.
6. To identify the best attractor, we then run individual Baum–Welch optimization for each potential attractor, thereby identifying the best values for the branch lengths c, the root prior *μ* and $\sigma^{2}$ and the transition parameters $\kappa_{i}$, $\phi_{i}$, and $\zeta_{i}$ for the full transition matrix (equation (18)). The final result, as relevant to the user, are the results with the transition matrix that resulted in the highest likelihood, which may either be the ladder-type matrix or one with a specific attractor state.

Once maximum likelihood estimates for the branch lengths c, the transition parameters $\kappa_{i}$, $\phi_{i}$, $\zeta_{i}$, and $a_{i}$ as well as the root prior *μ* and $\sigma^{2}$ are obtained, we infer state posterior probabilities $P(z_{l}|d,\theta )$ given the full data d and the learned parameters collectively denoted by θ, see Fig. 1c. We further determined the posterior mean migration rates as


(20) $${\bar{w}}_{il}=\sum_{j}w_{ij_{i}}P(z_{l}=j|d,\theta ).$$

To identify regions exhibiting either excess or dearth introgression compared to the genome-wide average, and are hence candidate regions to have experienced selection, we summarized these posterior probabilities as


$$\begin{array}{rl} P(z_{l}>a_{i}|d,\theta ) & =\sum_{j}I(j_{i}>a_{i})P(z_{l}|d,\theta ),\\ P(z_{l}<a_{i}|d,\theta ) & =\sum_{j}I(j_{i}<a_{i})P(z_{l}|d,\theta ), \end{array}$$

where I(⋅) denotes the indicator function. We then determined for each locus *l* the false discovery rates (FDRs) for excess [$q_{e}(l)$] and dearth [$q_{d}(l)$] introgression as


$$\begin{array}{rl} q_{e}(l) & =1-P(z_{l}>a_{i}|d,\theta ),\\ q_{d}(l) & =1-P(z_{l}<a_{i}|d,\theta ). \end{array}$$

### Implementation

We implemented the proposed inference scheme in C++ as a user-friendly command-line tool TreeSwirl. Our tool is open-source and available through a git-repository at bitbucket.org/wegmannlab/treeswirl, along with a documentation and a custom R package to visualize the results. Our implementation makes heavy use of the HMM framework of the statistical library stattools available at bitbucket.org/wegmannlab/stattools.

To streamline computations, we employ a straightforward clustering method to reduce the number of sampling size variance matrices $\Sigma_{l}$ that need to be considered:

1. We sort the vector of sample sizes according to the frequency of each occurrence.
2. To cluster, we identify the pair of vectors with the least occurrences and compute their weighted average.
3. We retain the weighted vector of sample sizes, remove the pair, and update the occurrence count as the sum of the deleted pair counts.
4. We repeat steps 1 to 3 until the desired number of $\Sigma_{l}$ is obtained.

Given a limited number *u* of such matrices and given that we use a finite number of discrete migration rates, there exist also an only finite number of matrices $S_{l}$ that can be precomputed in each Baum–Welch iteration to speed up the forward–backward pass through the HMM.

### Simulations

#### Simulations under Model Assumptions

**Fig. 2.**
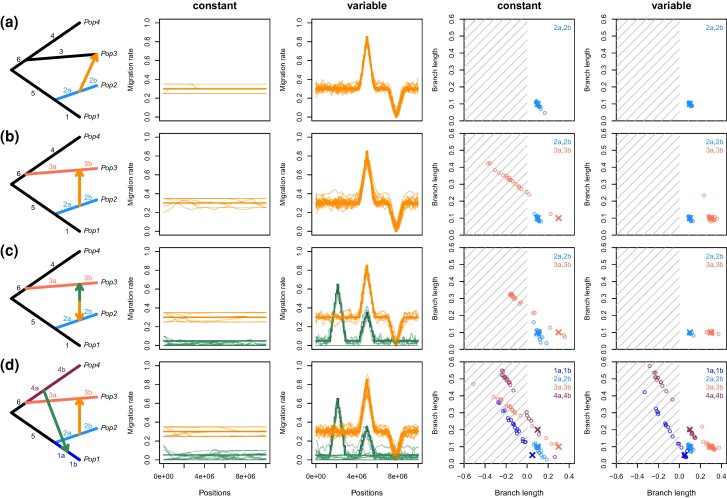
Performance of TreeSwirl as assessed by simulations under the TreeSwirl model. Shown are, for each of four cases of migration scenarios, the population graph (first column), estimates of locus-specific migration rates (posterior means, second and third column) and estimates of selected branch lengths (fourth and fifth column) for a case with variable (third and fifth column) and for a case without (second and fourth column) variation in migration rates along the simulated chromosome. Colors correspond to the migration edges and branch lengths of the same color in the population graph. Simulated migration rates are shown as a thick line and the estimates as a thin line per replicate. For branch lengths the simulated values are shown as a cross and estimates as open circles.

To demonstrate the power and limitations of our methods with respect to different demographic scenarios, we simulated data under the TreeSwirl model for population graphs of four populations under four different topologies, visualized in Fig. 2. In the first two cases, we designed a tree with a single migration event starting ancestral to Population 2 and directed to either Population 3 (Fig. 2a) or ancestral to Population 3 (Fig. 2b). The remaining two cases had two migration events, either bidirectional migration ancestral to the Populations 2 and 3 (Fig. 2c), or two unrelated events from ancestral of Population 2 and 4 to ancestral of Populations 3 and 1, respectively (Fig. 2d). For each tree, we simulated a single chromosome and considered both a scenario with a constant, genome-wide migration rate, as well as a scenario where the migration rate varied as shown in Fig. 2. For all cases, we simulated allele counts under the TreeSwirl model with N=100, μ=−0.5, $\sigma^{2}=0.3$, and J=21. We ran 20 replicates each.

#### fastsimcoal2

**Fig. 3.**
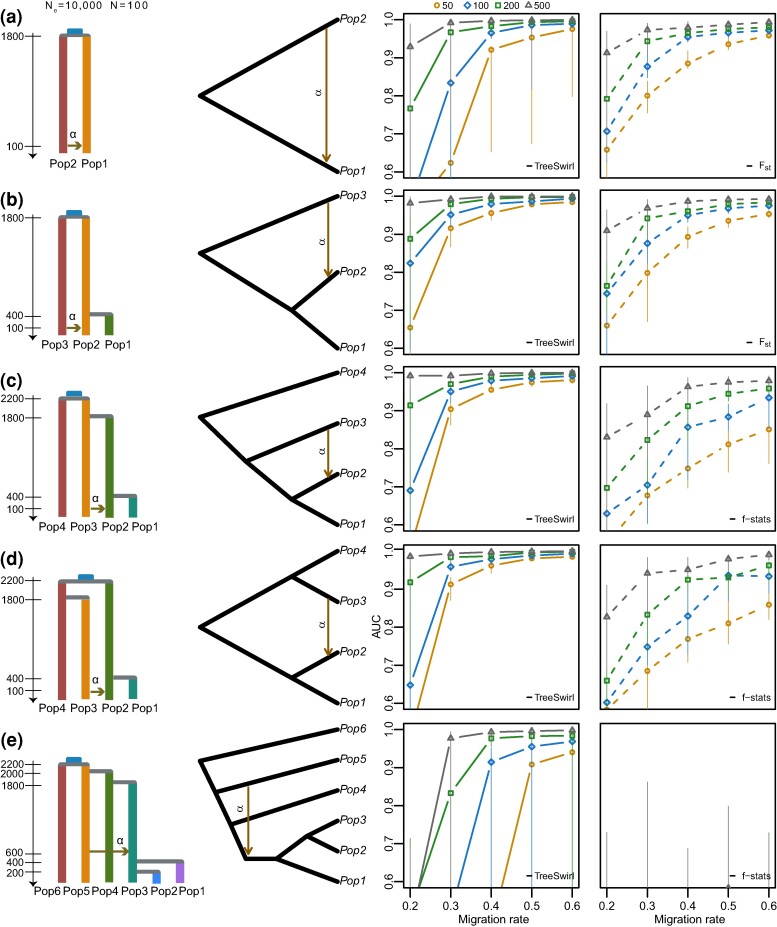
Performance of TreeSwirl and $f_{4}$-stats methods to measure the amount of introgression under different demographic histories with an background migration rate $\alpha_{b}=0.05$. First column: simulated demographic histories. Second column: schematic of the topology models. Third column: AUC measures for TreeSwirl to identify outlier loci (elevated migration compared to background rate) as a function of the peak width (symbols, in number of blocks) and peak migration (*x*-axis). Fourth column: AUC measure for the combination of summary statistic (*F_st_*, *D*- or *f*-related), window size and population subset (in case E only) that led to the highest AUC.

To compare TreeSwirl to competing methods, we used fastsimcoal2 (Excoffier et al. 2021) to simulate genomic data under seven different demographic scenarios only consisting of population splits and admixture pulses (but no population growth or continuous migration, Fig. 3). We maintained a constant effective population size of $N_{e}=10,000$ and used a sample size of N=100 for each population in all cases.

To simulate variation in admixture pulses along chromosomes, we simulated chromosomes of 10 Mb in length, each composed of independent DNA segments of length 1,000 bp with a per-locus recombination and mutation rate of $10^{-8}$, and a transition rate of 0.33. For each chromosome, we simulated 7,000 blocks with a background migration rate of 0.1, 1,000 blocks with the peak migration rate (either 0.2, 0.3, 0.4, 0.5, or 0.6) and 2,000 blocks with intermediate migration rate. The blocks with elevated migration rates were grouped into peaks, each consisting of *m* blocks at intermediate migration, *m* blocks at peak migration, and again *m* blocks at intermediate migration. We distributed the blocks evenly along the chromosome and consider peak width of m=50,100,200, and 500 blocks. In case we simulated two migration events, the second migration rates were constructed analogously but with all migration rates increased by 0.1 and the peaks arranged such that some peaks of the two migration events overlap fully, some partially and some not at all.

We generated ten replicates for each parameter combination and used a custom script to transform the generated output files into standard VCF (variant calling format) files and concatenating the 10,000 blocks corresponding to a single chromosome. Unless specified otherwise, we applied a minimum allele frequency filter of maf=0.05 with VCFtools (Danecek et al. 2011), resulting in a variable but rather low (0 to 3) number of polymorphic loci per block and thus resulted in data similar to what would be obtained after LD-pruning.

The filtered VCFs served as input for estimating sliding window $F_{st}$ for simulated data only consisting of two or three populations as well as for running D-suite Dinvestigate (Malinsky et al. 2021) with varying window sizes s=(10,50,100,150,200,250,300,350,400,450,500), a sliding locus of 1, and the true trio and corresponding outgroup for demographic scenarios with more than three populations. Concurrently, we executed TreeSwirl using the same filtered data and the expected tree topology.

We employed a receiver operating characteristic (ROC) curve analysis to assess the area under the curve (AUC), which summarizes the performance of the method in identifying loci with elevated migration rates. For the ROC analysis, we used the estimated mean posteriors obtained from TreeSwirl, along with the computed values of $F_{st}$, Patterson’s *D*, $f_{d}$, $f_{dM}$, and $d_{\;f}$, and compared them to the true migration rates. For each comparison, we used the statistics and window size that resulted in the best AUC.

### Real Data Application

We reanalyzed whole-genome sequencing from a recent study that identified putatively adaptive introgression from domestic goat into Alpine ibex (Münger et al. 2024). We restricted our analysis to species with at least four individuals: modern Alpine ibex (29 individuals), the Iberian ibex (four individuals), Bezoar ibex (six individuals), and domestic goats (16 individuals). We further focused on Chromosomes 23 on which significant introgression was previously reported for gene regions with immune-related genes such as MHC (Grossen et al. 2014; Münger et al. 2024), and included the flanking Chromosomes 21, 22, 24, and 25 for comparison. We used a VCF file provided by the authors. This file was previously filtered to a minimum minor allele frequency (MAF) of 0.05 and a maximal missingness of 0.9 across all samples studied in Münger et al. (2024), and was further thinned to have a minimal distance of 100 bp between consecutive SNPs (see Münger et al. 2024, for details). The admixture graph used was derived from the demographic model inferred by Münger et al. (2024), but we modeled two migration events starting ancestral to domestic goat and directed to Alpine ibex and one to Iberian ibex, respectively, since hybridization between domestic goats and Iberian ibex has also been reported (Cardoso et al. 2021). We used the physical positions of markers provided in the VCF file and set J=21.

## Results

### Simulations under the TreeSwirl Model

We conducted simulations under the above model to investigate the power of TreeSwirl to infer branch lengths and locus-specific migration rates. We focused on a tree of four populations and considered several cases of migration events of increasing complexity (Fig. 2) and simulated a case with and without variation in migration rates along the simulated chromosome.

The first case contains a single migration event from a population ancestral to Population 2 to Population 3 (Fig. 2a). In this case, the most challenging branch lengths to estimate are those leading to and from the source of the migration edge (branches 2a and 2b in Fig. 2a) since only their sum is relevant for the variance across loci in Population 2. However, additional information about their lengths comes from the covariance between Populations 2 and 3, and as a result their lengths as well as locus-specific migration rates are well estimated both when using a constant or a variable migration rate (Fig. 2a).

The second case is similar to the first, except that the migration edge now ends ancestral to Population 3 (Fig. 2b), leading to two pairs of branches that are challenging to infer: branches 2a and 2b and branches 3a and 3b (marked in blue and pink in Fig. 2b, respectively). As in the first case, information about the lengths of branches 2a and 2b stems from the variance of Population 2 and the covariance between Populations 2 and 3. In contrast, information about the lengths of branches 3a and 3b only stems from the variance of Population 3, and they are thus nonidentifiable in the case of constant migration with some branch lengths often inferred to be negative (Fig. 2b). This nonidentifiability issue has been previously observed by Pickrell and Pritchard (2012), and TreeMix consequently only allows for migration edges that end at tips or the end of branches. As shown in Fig. 2b, however, these branch lengths do become identifiable if loci vary in their migration rates and if that variation is explicitly modeled.

And as shown in Fig. 2c, variation in migration rates renders all branch lengths identifiably also in the case of bidirectional migration, which led to an accurate inference of locus-specific migration rates in all simulated replicates. While variation in migration rates renders all branch lengths identifiable also in the case of two migration events involving all four populations, our inference framework fails to identify the correct solutions in about two-thirds of all replicates (Fig. 2d). Closer inspection reveals that this mostly affects the branch lengths and migration rates related to the migration edge that is more ancient and has both lower average and less variability in migration rates. We note that these cases often result in negative branch lengths, which we found to be a good indicator that the population graph used has too many degrees of freedom for the data analyzed.

### Comparison to Related *D*- and *F*-Statistic Methods

**Fig. 4.**
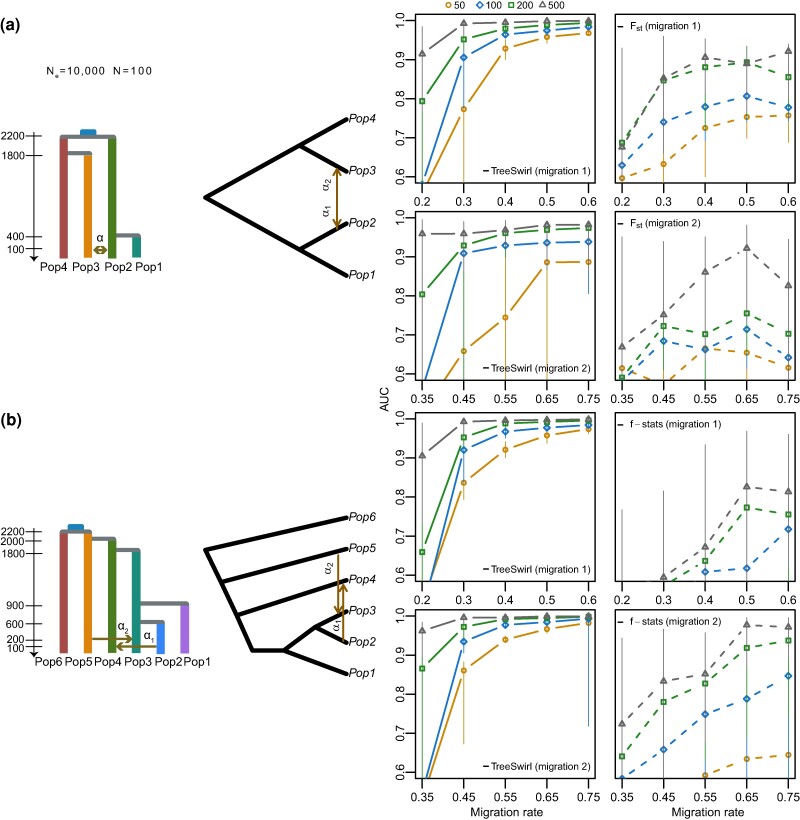
Performance of TreeSwirl to measure the amount of introgression under different demographic histories with two migration events. First column: simulated demographic histories. Second column: schematic of the topology models. Third and fourth columns: AUC measures to identify outlier loci (elevated migration compared to background rate) for migration edges $\alpha_{1}$ and $\alpha_{2}$, respectively, as a function of the peak width (symbols, in number of blocks) and peak migration (*x*-axis).

**Fig. 5.**
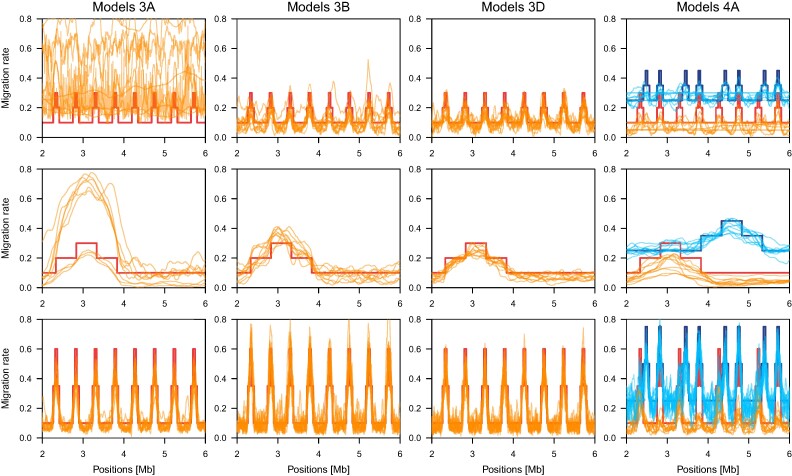
Accuracy of migration rate inference by TreeSwirl. Shown are the per-locus posterior mean migration rate estimates (thin lines) for the first 6 Mb (the first half of all simulated loci) obtained among ten replicates under four models (columns): Models A, B, and D from Fig. 3 and Model A from Fig. 4 (orange corresponds to the edge from Pop3 into Pop2, blue to the edge from Pop2 into Pop3). For each model, results are shown for three combinations of peak width and peak migration rate: 50 blocks and migration 0.3, 500 blocks and migration 0.3, and 50 blocks and migration 0.6. For Model 4A, the corresponding rate for the second migration event was 0.45, 0.45, and 0.75. In all cases, the simulated migration rate is shown as a thick line.

We generated coalescent simulations under seven demographic histories of population splits and mixtures outlined in Figs. 3 and 4. For each model, we used an effective population size of $N_{e}=10,000$, a sample size of N=100 and a shared common ancestor for all populations dating back ∼2,000 generations. We simulated a single chromosome composed of genomic blocks of 1,000 bp with different migration rates: 7,000 blocks were at a background migration rate, 1,000 blocks were at peak migration rate, and 2,000 blocks had an intermediate migration rate. We arranged these blocks to obtain a range of peak sizes and peak migration rates and for each case evaluated the power of TreeSwirl to identify regions with elevated migration rates (candidates for adaptive introgression, Figs. 3 and 4) and to infer the patterns of migration rates along the simulated genomes (Fig. 5). For the models involving a single migration rate, we compared the power of TreeSwirl to identify regions with elevated migration rates to the power of commonly used summary statistics, namely $F_{st}$ and *f*-statistics. We calculated these statistics using D-suite Dinvestigate (Malinsky et al. 2021) for various window sizes and, in case of more than four populations, for all population combinations possible. To render our comparison conservative, we then always kept the summary statistics, window size, and population combination resulting in highest power.

As shown in the third and fourth column of Fig. 3, TreeSwirl has overall higher power to identify regions with elevated migration rates than competing summary statistics, with a much lower false-positive rate, particularly if the background and elevated migration rates were rather similar (i.e. low peak migration rate). Importantly, TreeSwirl also had considerable power in identifying loci with elevated migration rates for models with two- and three-taxon topologies. This presents a significant advantage over $f_{4}$-stat methods, which are constrained to four-taxon configurations and require a predefined outgroup.

The most difficult scenario was the case of only two populations, in which TreeSwirl needs to estimate migration rates along four branch lengths. In the case of no variation in migration rates, the information in the data corresponds to only two variances and one covariance term, thus considerably less than the degrees of freedom in the model. Here, we simulated genomes with three different migration rates, but still no accurate inference of migration rates was possible in case of a peak width of 50 blocks and a peak migration of 0.3 with estimates of individual replicates varying across the entire range of possible values (Fig. 5, Model 2A). When using a larger peak width of 500 blocks (but still a peak migration of 0.3), TreeSwirl generally inferred the proper pattern in migration rate variation, but overestimated the peak migration considerably in several replicates. When using a higher peak migration rate of 0.6 (but still a peak width of 50 blocks), estimates were much more accurate, even if the peak migration rate were slightly underestimated. Consequently, the power to identify regions with elevated migration rates was higher than for *F_st_* in these cases (Fig. 3a).

As more populations are included, the number of variances and covariances increases faster than the number of branch lengths (M(M+1)/2 vs. 2M−1 for a strictly bifurcating graph without migration), rendering model parameters easier to infer (but see Fig. 2 for how migration edges complicate things). Consequently, TreeSwirl also inferred migration rates much more accurately for simulations conducted with more populations (Fig. 5, Models 3B and 3D), although it remained more difficult in the case of very short peak width and low peak migration rates. Nonetheless, in these cases TreeSwirl showed higher power to identify regions with elevated migration rates than even the best of all summary statistics tested (Fig. 3b to d).

Noteworthy is the model illustrated in Fig. 3e, which included many populations but turned out to be challenging for inference. In that scenario, the migration event occurred a relatively long time after the involved population split, and none of the other populations sheds additional information on the allele frequencies at the time of migration, rendering it difficult to distinguish migration from pure drift. Since the populations Pop1 and Pop2 branch of after the migration event only, the scenario is actually very similar to that explored in Fig. 2b, which we found only to be identifiable with sufficient variation in migration rates. Interestingly, and despite its rather low power, TreeSwirl still outperformed all summary statistics tested in identifying loci with elevated migration rates also under this model.

We next investigated the power of TreeSwirl to identify loci with elevated migration rates under two demographic models involving two migration edges. The first model, shown in Fig. 4a, extends the single migration, four-population model shown in Fig. 3c with bidirectional gene flow. For the migration edge from Population 3 into Population 2 (migration 1 in Fig. 4a), migration rates were rather accurately inferred (Fig. 5, Model 4A in blue) and consequently the power to identify loci with elevated migration rates was only slightly reduced compared to the single migration case (left panel in Fig. 3c). In contrast, inferring migration rates was more challenging for the migration edge from Population 2 into Population 3 due to the rather deep split between Populations 3 and 4 (Fig. 5 Model 4A in orange). Consequently, the power to identifying such loci was considerably lower for that migration edge (migration 2 in Fig. 4a). The second model, shown in Fig. 4b, is similar to the single migration model shown in Fig. 3e, but with two recent edges involving different branches, for both of which the power to identify loci with elevated migration rates was comparable to that for single population models. Notably, TreeSwirl had higher power to detect loci with elevated migration rates than any of the summary statistics tested in all cases, likely because it could make use of all population samples jointly while the summary statistics were limited to a subset of comparisons only. In the first model, for instance, only *F_st_* between population pairs could be used to distinguish between the two migration edges. Similarly, in the second model, data from Pop3 could not be used to form $f_{4}$-statistics to identify loci with elevated loci for migration edge 1 from Pop2 into Pop4, although that population would be most informative in the absence of migration edge 2.

### MAF Filtering and Runtime Considerations

**Fig. 6.**
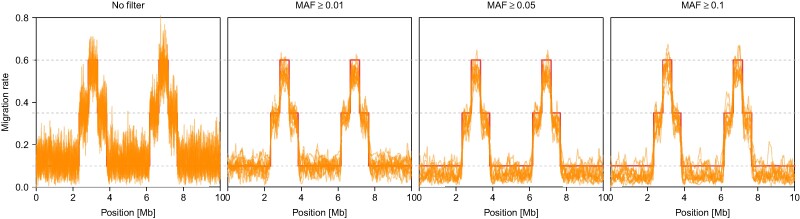
Effect of filtering on MAF. Shown are the per-locus posterior mean migration rate estimates (thin lines) for the simulations of Model D from Fig. 3 with a peak width of 500 blocks and peak migration of 0.6 (thick lines), for no MAF filter and for only keeping loci with MAF ≥0.01, MAF ≥0.05, and MAF ≥0.1.

Across all simulations we noted that migration rates tended to rather be under- than overestimated (Fig. 5), which we found to be a direct consequence of filtering for minimum MAF. We illustrate this in Fig. 6 for the model from Fig. 3d and the easiest case (peak width of 500 blocks, peak migration of 0.6): with filtering out low frequency variants more strictly, per-locus migration estimates tend to get lower. The reason for this is likely that low frequency variants, which are often population-specific, are particularly informative about migration, while high frequency variants tend to be shared among populations regardless of migration. Although this may suggest that no MAF filter should be used, we note that low frequency variants also lead to more noisy estimates of per-locus migration rates (Fig. 6a) and considerably longer run times, which scales linearly with the number of loci.

Additional factors affecting the computational costs of running TreeSwirl include: (i) the number of discrete states *J*, with which runtime scales quadratically, (ii) the number of migration edges, with which runtime scales exponentially, and (iii) the number of matrices $\Sigma_{l}$, with which runtime scales linearly. Computations may be efficiently distributed across multiple computer nodes by dividing the genome into independent segments, such as individual chromosomes or chromosome arms. This approach is valid because linkage does not persist across chromosome boundaries and is typically weak across the centromere.

### Introgression from Domestic Goats into Alpine Ibex

**Fig. 7.**
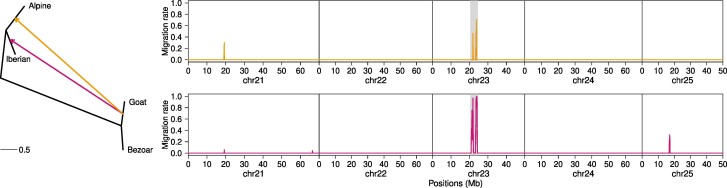
Inference of introgressed loci in Alpine and Iberian ibex. Shown is the inferred population graph along with inferred migration rates (posterior means) along Chromosomes 21 to 25 for Alpine ibex (top, edge from Goat to Alpine) and Iberian ibex (bottom, edge from Goat to Iberian). The region reported by Münger et al. (2024) on Chromosome 23 is marked with a gray background.

Alpine ibex are a wild goat species native to the European alps. It suffered from a near-extinction two centuries ago, but has since recovered thanks to repopulation efforts. It is known that Alpine ibex occasionally hybridize with domestic goats and adaptive introgression has been reported for several genomic regions, particularly for a region on Chromosome 23 that contains immune-related genes such as MHC (Grossen et al. 2014; Münger et al. 2024). Here, we used TreeSwirl to reanalyze whole-genome sequencing data of Alpine ibex, Iberian ibex, Bezoar ibex, and domestic goat to quantify locus-specific rates of introgression from domestic goat into Alpine and Iberian ibex along Chromosomes 21 to 25. As shown in Fig. 7, we generally inferred very low migration rates from domestic goat into Alpine ibex with an inferred attractor state at zero. Nonetheless, we identified a clear signal of introgression on Chromosomes 23 between 20 and 24 Mb, corresponding well to the region previously reported (Münger et al. 2024) and containing MHC. We also identified an additional, smaller region of introgression on Chromosome 21.

We also inferred rather low overall migration rates from domestic goat into Iberian ibex, again with the attractor state estimated at zero. Interestingly, the region on Chromosome 23 inferred as introgressed in Alpine ibex was also inferred as strongly introgressed in Iberian ibex, this time along an additional, smaller region on Chromosome 25. Since the two ibex species likely diverged prior to the domestication of goats, the signal on Chromosome 23 is thus indicative of independent adaptive introgression in both ibex species. But we note that given the frequency dependent selection likely acting on the MHC region, the signal may also be indicative of shared ancient polymorphisms among all goat species, although the high migration rates we inferred (close to 1.0) suggest this scenario to be less likely.

## Discussion

One approach to infer historical relationships among populations is to model allele frequency changes along a phylogenetic tree as a Gaussian process (Cavalli-Sforza and Edwards 1967; Felsenstein 1981). This rather old concept was recently revived by extending the model to a graph with migration edges and by providing a user-friendly tool TreeMix to infer parameters under such a graph (Pickrell and Pritchard 2012). However, the TreeMix model assumes migration rates to be constant along the genome, an assumption that may not hold in the face of selection or strong genetic drift. Indeed, theory predicts variation in the rate of effective gene flow along the genome (Harrison 1993), in which local barriers to gene flow are anticipated to emerge from the random accumulation of Dobzhansky–Muller incompatibilities, both under models of secondary contact after isolation (Barton and Gale 1993) as well as under models of continuous gene flow during speciation (Wu 2001). In the case of gene flow between highly divergent gene pools, selection is likely to act as the primary driving force for variation in effective gene flow along the genome, with rates of introgression being particularly low in genomic regions involved in adaptation, so called islands of speciation, but potentially much higher in regions free from the selection pressure (Dasmahapatra et al. 2012).

In light of these considerations, we here present TreeSwirl, an extension of the model described in Pickrell and Pritchard (2012) that allows for mixture proportions to vary along the genome in an auto-correlated way that reflects the effect of linkage. Using simulations, we found the power of TreeSwirl to detect loci with elevated gene flow to be a function of several parameters: First, the power generally increases with the difference between the peak and background migration rate as well as with the size of the region affected by elevated gene flow (and hence the local recombination structure). Second, the power generally increased if data from additional populations was availably, ideally populations that she a lot of information about the allele frequencies at the time of the migration events such as population that split of right before a population received migrants. Third, sufficient variation in migration rates along the genome is required to accurately infer some branch lengths, which in turn increases power to infer outlier loci. Finally, and although untested here, we expect sample size to affect power as well as low sample sizes lead to a lot of uncertainty regarding the current allele frequencies. As our simulations indicated, TreeSwirl had high power to infer even relatively short regions of 50 kb in many scenarios, and may identify even much shorter regions under a favorable population graph and provided that peak (or trough) migration rates was substantially different from the background rate.

We compared the power of TreeSwirl to existing methods related to *D*- and *f*-statistics, such as $F_{st}$, Patterson’s *D* (Patterson et al. 2012), $f_{d}$ (Martin et al. 2015), $f_{dM}$ (Malinsky et al. 2015), and $d_{\;f}$ (Pfeifer and Kapan 2019), which have been frequently applied to identify signatures of introgression using arbitrary genomic window sizes. As our simulations showed, TreeSwirl had superior accuracy and sensitivity in detecting retrogressed loci under all demographic histories investigated. The approach presented here also addresses numerous constraints inherent to the use of related *D*- and *f*-statistics. First, these summary statistics are limited to bifurcating four-population topologies. In cases involving graphs of five or more populations, the simplest option is to subsample a section of the graph in the appropriate configuration, as done in D-suite (Malinsky et al. 2021) used here. In cases involving two- or three-population topologies, one would need to resort to *F_st_*-based metrics. In contrast, the method presented here is not constraint by topology, working well with any number of populations and also under topologies that include polytomies.

Second, our HMM-based approach to model linkage eliminates the need to specify window sizes. Instead, the parameters governing auto-correlation are directly inferred from the data along with introgression rates. In our simulations, the choice of window sizes, as well as the choice of the specific statistics to use, had a big impact on power. To ensure a fair comparison between methods, we thus tested all available summary statistics for a wide range of window sizes and only report the results of the combination of summary statistics and window size that was optimal for each individual case. In applications to real data, however, such explorations are not possible, likely leading to an even larger difference in power between TreeSwirl and these summary statistics.

Third, TreeSwirl supports graphs with multiple migration edges for which introgression rates are learned simultaneously. However, it is important to note that the performance of TreeSwirl is likely dependent on the quality of the tree topology used as input and may not perform well if the tree topology is poorly resolved or incorrect. Similarly, we caution that TreeSwirl just as TreeMix assumes that population allele frequencies can be explained through a population graph with splits, drift along branches and potential mixtures. While this approach has been proved useful in many cases, it is easy to think of scenarios that violate these assumptions. One such scenario consists of populations that have diverged long enough to hardly share any polymorphisms. This leads to negative covariance between populations (the presence of a mutation in one population implies its absence in the others), which cannot be captured by a population graph. While experimenting with such cases showed TreeSwirl to be remarkably robust, it should not be confused with phylogenetic approaches.

Other scenarios that violate the underlying model assumptions are cases of extensive continuous gene flow as well as cases of population structure not well captured with a graph. Such scenarios were shown to lead to spurious inference of introgression using other methods (e.g. Eriksson and Manica 2014; Lawson et al. 2018; Tournebize and Chikhi 2023), and while we have not explored these scenarios in detail here, we are convinced they can lead to spurious inference by both TreeSwirl and TreeMix and appeal to users to carefully assess model assumptions of these any other tools they use. Nonetheless, and as we showed through both simulations and a data application, the model introduced here is powerful to identify introgressed loci under many scenarios and will thereby contribute to a better understanding of the role of introgression in evolution, which is expected to act as a major driver in adaptation to ongoing global changes (Suarez-Gonzalez et al. 2018).

## Supplementary Material

msae137_Supplementary_Data

## Data Availability

The authors affirm that all data required to validate the conclusions of this article are either included within the article itself or accessible through the indicated repositories. The source code for TreeSwirl can be found in the following Git repository: bitbucket.org/wegmannlab/treeswirl2, which also contains a user manual. This study did not generate any new data.
