# Supplementary material for: Inference of Locus-Specific Population Mixtures from Linked Genome-Wide Allele Frequencies
Source: Mol Biol Evol. 2024 Jul 3;41(7):msae137. doi: 10.1093/molbev/msae137 (PMC11255385; doi:10.1093/molbev/msae137)
Supplement: msae137_Supplementary_Data [file msae137_supplementary_data.pdf]

Supplemental Information: Inference of Locus-Specific Population Mixtures From Linked Genome-Wide Allele Frequencies

Carlos S. Reyna-Blanco[a,b,c], Madleina Caduff[a,b,c], Marco Galimberti[d], Christoph Leuenberger[e], and Daniel Wegmann[b,c,*]

[a]These authors contributed equally
[b]Department of Biology, University of Fribourg, 1700 Fribourg, Switzerland
[c]Swiss Institute of Bioinformatics, 1700 Fribourg, Switzerland
[d]Yale School of Medicine, New Haven, CT
[e]Department of Mathematics, University of Fribourg, 1700 Fribourg, Switzerland
[*]Corresponding author, daniel.wegmann@unifr.ch

Contents

1 Inference **S1**
1.1 Optimization of Emission Probabilities . . . S2
1.2 Optimization of Transition Probabilities . . . S3

2 Initialization **S4**
2.1 Step 1: A Gaussian Mixture Model . . . S4
2.1.1 Inference . . . S5
2.1.2 Initial guess and choice of number of classes . . . S6
2.2 Step 2: Guessing initial branch length . . . S6

3 References **S7**

1 Inference

We have to infer the transition and emission probabilities from the observations. Since we do not know through which hidden states the trajectories went, we apply the Baum-Welch algorithm to estimate the following parameters $\boldsymbol{\theta}$ of the HMM:

1. the initial state distribution $\eta_j = \mathbb{P}(z_1 = j)$;
2. the transition matrix parameters, where i is associated to each migration edge:

   κ_i (scaling factors),
   ϕ_i (control number of sites not in "neutral state"),
   ζ_i (control extent of sites not in "neutral state"),
   a_i (attractors, "neutral states");

3. the branch lengths $\boldsymbol{c} = (c_1, \ldots, c_k)$;
4. the parameters μ, σ^2 of the root state prior.

Applying the Baum-Welch algorithm to HMM is rather standard (Baum et al., 1970), but it involves lengthy calculations for this rather complex model, as we will show in the following. The Baum-Welch algorithm is a variant of the Expectation-Maximization (EM) algorithm which updates the parameters

by alternating between an E-step and an M-step. Let $\boldsymbol{\theta}'$ denote the old and $\boldsymbol{\theta}$ the new parameters. In the E-step of the algorithm, we calculate the expected complete data log-likelihood given by

$$\begin{aligned} Q(\boldsymbol{\theta}, \boldsymbol{\theta}') = & \sum_j \gamma_1(j) \log \eta_j + \sum_l \sum_{j,j'} \gamma_l(j,j') \log \mathbb{P}_l(j,j') \\ & + \sum_l \sum_j \gamma_l(j) \log \pi(\mathbf{d}_l | \mu, \sigma^2, \mathcal{G}_j), \end{aligned} \tag{S.1}$$

where we used the notation from the main text and where

$$\gamma_l(j) = \mathbb{P}(z_l = j \mid \mathbf{d}_{1:L}, \boldsymbol{\theta}')$$

and

$$\gamma_l(j,j') = \mathbb{P}(z_{l-1} = j, z_l = j' \mid \mathbf{d}_{1:L}, \boldsymbol{\theta}').$$

These γ's can be calculated by the standard forward-backward algorithm for HMMs (Murphy, 2012). Maximization w.r. to the new parameters is standard for the initial state distribution: $\eta_j^* = \gamma_1(j)$. For each EM iteration, we also need to store all γ's to calculate the derivatives shown below.

1.1 Optimization of Emission Probabilities

To infer the branch lengths, we use the Newton-Raphson (NR) algorithm (Nocedal and Wright, 2006; Lange, 2010). To avoid boundary issues, we allow for negative branch lengths as long as they result in valid variance-covariance matrices $\boldsymbol{W}_j$. Specifically, we check that i) all diagonal entries of $\boldsymbol{W}_j$ are ≥ 0 and that ii) the matrix is positive semi-definite. We do so for all boundary cases, i.e. for the $\boldsymbol{W}_j$ of all combinations with either zero or full migration on each migration edge (e.g. (0,0), (0,1), (1,0) and (1,1) in case of two migration edges). However, we output a warning whenever negative branch lengths are inferred as they are often a sign of a misspecified tree.

The gradient of $Q(\boldsymbol{\theta}, \boldsymbol{\theta}')$ w. r. to the branch lengths is

$$\frac{\partial}{\partial c_k} Q(\boldsymbol{\theta}, \boldsymbol{\theta}') = \sum_l \sum_j \gamma_l(j) \frac{\partial}{\partial c_k} \log \pi(\mathbf{d}_l | \mu, \sigma^2, \mathcal{G}_j). \tag{S.2}$$

The terms $\frac{\partial}{\partial c_k} \log \pi(\mathbf{d}_l | \mu, \sigma^2, \mathcal{G}_j)$ can be evaluated by defining the matrix $\boldsymbol{J}_{jk}$ given by (6) . (Note that there we did not yet have the index j because at this stage the matrix was defined for the value w instead of w_j.) From (7) we get:

$$\frac{\partial}{\partial c_k} \boldsymbol{S}_{lj} = \frac{\partial}{\partial c_k} \boldsymbol{W}_j = \boldsymbol{J}_{jk}.$$

We can now calculate the partial derivatives of all the terms in (15). The derivative of the identity $\boldsymbol{S}_{lj}^{-1} \boldsymbol{S}_{lj} = \boldsymbol{I}$ yields

$$\frac{\partial}{\partial c_k} \boldsymbol{S}_{lj}^{-1} = -\boldsymbol{S}_{lj}^{-1} \boldsymbol{J}_{jk} \boldsymbol{S}_{lj}^{-1}.$$

Using Jacobi's formula we get

$$\frac{\partial}{\partial c_k} \log \det \boldsymbol{S}_{lj} = \mathrm{tr} \left(\boldsymbol{S}_{lj}^{-1} \frac{\partial}{\partial c_k} \boldsymbol{S}_{lj} \right) = \mathrm{tr} \left(\boldsymbol{S}_{lj}^{-1} \boldsymbol{J}_{jk} \right).$$

With this information we obtain the partial derivatives

$$\begin{aligned} \frac{\partial}{\partial c_k} \log \pi(\mathbf{d}_l | \mu_l, \mathcal{G}_j) = & -\frac{1}{2} \mathrm{tr} \left(\boldsymbol{S}_{lj}^{-1} \boldsymbol{J}_{jk} \right) \\ & + \frac{1}{2} (\mathbf{d}_l - \mu_l \mathbf{1})' \boldsymbol{S}_{lj}^{-1} \boldsymbol{J}_{jk} \boldsymbol{S}_{lj}^{-1} (\mathbf{d}_l - \mu_l \mathbf{1}) \end{aligned} \tag{S.3}$$

that form a gradient function of the branch lengths:

$$\boldsymbol{F}(\boldsymbol{c}) = \boldsymbol{F}(c_1, \ldots, c_K) := \left(\frac{\partial}{\partial c_1} Q(\boldsymbol{\theta}, \boldsymbol{\theta}'), \ldots, \frac{\partial}{\partial c_K} Q(\boldsymbol{\theta}, \boldsymbol{\theta}') \right)'.$$

As mentioned, we will use the NR algorithm to solve $\boldsymbol{F}(\boldsymbol{c}) = (0, \ldots, 0)'$, for which we also need the Jacobian of $\boldsymbol{F}$. From (eq. S.2) and (eq. S.3) we obtain

$$\frac{\partial}{\partial c_i}\boldsymbol{F}_k(\boldsymbol{c}) = \frac{1}{2}\sum_l \sum_j \gamma_l(j)\Big[\mathrm{tr}\boldsymbol{K}_{lj,ik} - (\mathbf{d}_l - \mu_l \mathbf{1})' \left(\boldsymbol{K}_{lj,ik} + \boldsymbol{K}_{lj,ki}\right) \boldsymbol{S}_{lj}^{-1}(\mathbf{d}_l - \mu_l\mathbf{1})\Big], \tag{S.4}$$

where we introduce the notation

$$\boldsymbol{K}_{lj,ik} = \boldsymbol{S}_{lj}^{-1}\boldsymbol{J}_{ji}\boldsymbol{S}_{lj}^{-1}\boldsymbol{J}_{jk}.$$

The parameter μ of the root state prior can be inferred explicitly if we know σ^2. We get

$$\mu * = \frac{\sum_l \sum_j \gamma_l(j)\mathbf{1}'\boldsymbol{S}_{lj}^{-1}\mathbf{d}_l}{\sum_l \sum_j \gamma_l(j)\mathbf{1}'\boldsymbol{S}_{lj}^{-1}\mathbf{1}} \tag{S.5}$$

where we have set $\boldsymbol{S}_{lj} = \boldsymbol{\Sigma}_l + \boldsymbol{W}_j + \sigma^2\mathbf{1}\mathbf{1}'$, see Eq. (14).

There is no simple formula for σ^2 and we optimize for it with the Newton-Raphson algorithm. To this end, we need the first and second derivatives of (eq. S.1) with respect to σ^2. Using Jacobi's formula, we obtain the expression

$$\frac{\partial}{\partial \sigma^2}Q(\boldsymbol{\theta}, \boldsymbol{\theta}') = -\frac{1}{2}\sum_{j,l}\gamma_l(j)\left[\mathrm{tr}(\boldsymbol{T}_{lj}) - (\mathbf{d}_l - \mu\mathbf{1})'\boldsymbol{T}_{lj}\boldsymbol{S}_{lj}^{-1}(\mathbf{d}_l - \mu\mathbf{1})\right],$$

wherein we write $\boldsymbol{T}_{lj} = \boldsymbol{S}_{lj}^{-1}\mathbf{1}\mathbf{1}'$. For the second derivative of Q w.r. to σ^2 we get

$$\frac{\partial^2}{(\partial\sigma^2)^2}Q(\boldsymbol{\theta}, \boldsymbol{\theta}') = \frac{1}{2}\sum_{j,l}\gamma_l(j)\left[\mathrm{tr}(\boldsymbol{T}_{lj}^2) - 2(\mathbf{d}_l - \mu\mathbf{1})'\boldsymbol{T}_{lj}^2\boldsymbol{S}_{lj}^{-1}(\mathbf{d}_l - \mu\mathbf{1})\right].$$

1.2 Optimization of Transition Probabilities

The proposed transition matrix is parametrized by κ_i, ϕ_i and ζ_i, and additionally by an index a_i that denotes the row index of the attractor (0 corresponding to the bottom row, 1 to the second row, and so on):

$$\boldsymbol{\Lambda}_i = \begin{pmatrix} -1 & 1 & 0 & 0 & \ldots & 0 & 0 & 0 & 0 \\ 1-\zeta_i & -2 & 1+\zeta_i & 0 & \ldots & 0 & 0 & 0 & 0 \\ 0 & 1-\zeta_i & -2 & 1+\zeta_i & \ldots & 0 & 0 & 0 & 0 \\ \vdots & \vdots & \vdots & \vdots & \ddots & \vdots & \vdots & \vdots & \vdots \\ 0 & 0 & 0 & 0 & \ldots & 1+\zeta_1 & -2 & 1-\zeta_i & 0 \\ 0 & 0 & 0 & 0 & \ldots & 0 & 1+\zeta_i & -2 & 1-\zeta_i \\ 0 & 0 & 0 & 0 & \ldots & 0 & 0 & 1 & -1 \end{pmatrix}$$

with the attractor row given by

$$\begin{pmatrix} 0 & \ldots & 0 & \phi_i & -2\phi_i & \phi_i & 0 & \ldots & 0 \end{pmatrix}.$$

The parameter ζ_i models the rate of moving away from the periphery towards the attractor. If $\zeta_i = 0$, it's equally likely to move up or down. If $\zeta_i = 1$, it is impossible to move to the periphery. The parameter ϕ_i models the rate of leaving the attractor. If $\phi_i = 0$, it's impossible to leave the attractor. If $\phi_i = 1$, the rate of leaving the attractor matches that of leaving any other state.

If there are only three states, the generating matrix reduces to:

$$\mathbf{\Lambda}_i = \begin{cases} \kappa_i \cdot \begin{pmatrix} -\phi_i & \phi_i & 0 \\ 1+\zeta_i & -2 & 1-\zeta_i \\ 0 & 1 & -1 \end{pmatrix} & \text{if } a_i = 0 \\ \kappa_i \cdot \begin{pmatrix} -1 & 1 & 0 \\ \phi_i & -2\phi_i & \phi_i \\ 0 & 1 & -1 \end{pmatrix} & \text{if } a_i = 1 \\ \kappa_i \cdot \begin{pmatrix} -1 & 1 & 0 \\ 1-\zeta_i & -2 & 1+\zeta_i \\ 0 & \phi_i & -\phi_i \end{pmatrix} & \text{if } a_i = 2. \end{cases}$$

Note that in the case of three states and $a_i = 1$ there is no ζ_i.

The stationary is complicated to calculate explicitly and we obtain it by solving a system of normal equations. For optimizing the transition matrix parameters κ_i, ϕ, ζ_i for a specific a_i, we use the Nelder-Mead (Nelder and Mead, 1965) algorithm.

2 Initialization

As the Baum-Welch algorithm is sensitive to good starting values, we developed a multi-step strategy to initialize the branch lengths $\boldsymbol{c}$, the root priors μ and σ_2 as well as the transition matrix parameters to reasonable values.

2.1 Step 1: A Gaussian Mixture Model

In the first step, we seek to get a good estimate of the variance-covariance matrices $\boldsymbol{W}_l$ without imposing any constraints given by the graph $\mathcal{G}$. To account for possible variation in $\boldsymbol{W}_l$ across loci due to variation in migration strengths, we partition the loci into R classes and infer the variance-covariance matrices $\mathbf{W}_r$ across populations for each class $r = 1, \ldots, R$.

We proceed as follows: We assume that the ancestral frequencies μ_l, $l = 1, \ldots, L$, are drawn i.i.d. from the prior $\mathcal{N}(\mu_0, \sigma_0^2)$ and that the observations are the random vectors $\mathbf{d}_l$ that are drawn from a series of Gaussian mixture models (GMM) with density

$$\mathbf{d}_l \sim \sum_{r=1}^{R} \pi_r \mathcal{N}(\mathbf{d}_l | \mu_l \mathbf{1}, \mathbf{W}_r), \quad l = 1, \ldots, L,$$

where we use the abbreviation

$$\mathcal{N}(\mathbf{d}_l | \mu_l \mathbf{1}, \mathbf{W}_r) = \frac{1}{\sqrt{(2\pi)^M |\mathbf{W}_r|}} \exp\left[-\frac{1}{2} (\mathbf{d}_l - \mu_l \mathbf{1})' \mathbf{W}_r^{-1} (\mathbf{d}_l - \mu_l \mathbf{1}) \right].$$

That is, the values $\mathbf{d}_l$ are drawn with probabilities (mixing weights) π_r from one of the R classes each of which produces random vectors from a multivariate normal distribution with mean $\mu_l \mathbf{1}$ and variance matrix $\mathbf{W}_r$. Clearly

$$\sum_{r=1}^{R} \pi_r = 1.$$

The parameters of this model are thus:

1. the variance matrices $\mathbf{W}_r$, $r = 1, \ldots, R$, for each of the R classes of the GMM;
2. the mean μ_0 and the variance σ_0^2 of the Gaussian prior for μ_l;
3. the mixing weights π_r.

To simplify the notation we will call this whole set of parameters $\boldsymbol{\theta}$.

2.1.1 Inference

We apply the EM algorithm in order to estimate these parameters. The hidden data for the EM algorithm are:

1. The mean vector $\boldsymbol{\mu} = (\mu_1, \ldots, \mu_L)$. These are continuous numbers that will be integrated out in the E-step.

2. The vector $\boldsymbol{s} = (s_1, \ldots, s_L)$ that indicates for each site which of the classes has been chosen.

E-step. The complete data likelihood is given by

$$L_c(\boldsymbol{\theta}; \mathbf{d}, \boldsymbol{\mu}, \boldsymbol{s}) = \prod_{l=1}^{L} \sum_{r=1}^{R} \operatorname{Ind}(r = s_l)\ \mathcal{N}(\mathbf{d}_l | \mu_l \mathbf{1}, \mathbf{W}_r)\ \frac{1}{\sqrt{2\pi}\sigma_0} e^{-\frac{1}{2\sigma_0^2}(\mu_l - \mu_0)^2}.$$

The expected complete data log-likelihood is calculated as

$$Q(\boldsymbol{\theta}; \boldsymbol{\theta}') = \mathbb{E}\left[\log L_c(\boldsymbol{\theta}; \mathbf{d}, \boldsymbol{\mu}, \boldsymbol{s}) | \boldsymbol{\theta}'\right]. \tag{S.6}$$

Using Bayes rule for linear Gaussian systems (see Murphy, 4.4.1), it is not hard to check that

$$\pi(\mu_l | \mathbf{d}_l, r, \boldsymbol{\theta}') = \mathcal{N}(\nu_{lr}, \rho_r^2)$$

where

$$\nu_{lr} = \frac{\mathbf{1}' \mathbf{W}_r'^{-1} \mathbf{d}_l + \mu_0' / \sigma_0'^2}{\mathbf{1}' \mathbf{W}_r'^{-1} \mathbf{1} + 1/\sigma_0'^2}, \quad \rho_r^2 = \frac{1}{\mathbf{1}' \mathbf{W}_r'^{-1} \mathbf{1} + 1/\sigma_0'^2}. \tag{S.7}$$

In order to calculate the expectation in (eq. S.6) we need the values $p'_{lr} = \mathbb{P}(r | \mathbf{d}_l, \boldsymbol{\theta}')$ which by Bayes' theorem are given by

$$p'_{lr} = \frac{\pi'_r \mathcal{N}\left(\mathbf{d}_l | r, \mu'_0, {\sigma_0}'^2\right)}{\sum_{s=1}^{R} \pi'_s \mathcal{N}\left(\mathbf{d}_l | s, \mu'_0, {\sigma_0}'^2\right)}$$

where

$$\mathcal{N}\left(\mathbf{d}_l | r, \mu'_0, {\sigma_0}'^2\right) = \mathcal{N}\left(\mathbf{d}_l \mid \mu'_0 \mathbf{1}, \mathbf{W}'_r + {\sigma_0}'^2 \mathbf{1}\mathbf{1}'\right).$$

M-step. First we maximize with respect to $\mathbf{W}_r$. Writing only the relevant part for the optimization, we get for the expected complete data log-likelihood

$$Q_1(\boldsymbol{\theta}; \boldsymbol{\theta}') = -\frac{1}{2} \sum_{l=1}^{L} \sum_{r=1}^{R} p'_{lr} \left(\log |\mathbf{W}_r| + \int_{-\infty}^{\infty} (\mathbf{d}_l - \mu_l \mathbf{1})' \mathbf{W}_r^{-1} (\mathbf{d}_l - \mu_l \mathbf{1})\ \pi(\mu_l | \mathbf{d}_l, r, \boldsymbol{\theta}')\ \mathrm{d}\mu_l \right).$$

Using the well-known law for the matrix derivative

$$\frac{\partial}{\partial \boldsymbol{W}^{-1}} \log |\boldsymbol{W}| = -\frac{\partial}{\partial \boldsymbol{W}^{-1}} \log |\boldsymbol{W}^{-1}| = -\boldsymbol{W},$$

we obtain

$$\frac{\partial}{\partial \mathbf{W}_r^{-1}} Q_1(\boldsymbol{\theta}'; \boldsymbol{\theta}') = \frac{1}{2} \sum_{l=1}^{L} p'_{lr} \left(\mathbf{W}_r - \int_{-\infty}^{\infty} (\mathbf{d}_l - \mu_l \mathbf{1})(\mathbf{d}_l - \mu_l \mathbf{1})'\ \pi(\mu_l | \mathbf{d}_l, r, \boldsymbol{\theta}')\ \mathrm{d}\mu_l \right).$$

Setting this expression equal to zero, we get the optimal value

$$\mathbf{W}_r^* = \frac{1}{p'_r} \sum_{l=1}^{L} p'_{lr} \int_{-\infty}^{\infty} (\mathbf{d}_l - \mu_l \mathbf{1})(\mathbf{d}_l - \mu_l \mathbf{1})'\ \pi(\mu_l | \mathbf{d}_l, r, \boldsymbol{\theta}')\ \mathrm{d}\mu_l.$$

where $p'_r = \sum\limits_{l=1}^{L} p'_{lr}$. Using

$$\int_{-\infty}^{\infty} \mu_l\ \pi(\mu_l | \mathbf{d}_l, r, \boldsymbol{\theta}')\ \mathrm{d}\mu_l = \nu_{lr} \tag{S.8}$$

and

$$\int_{-\infty}^{\infty} \mu_l^2 \; \pi(\mu_l|\mathbf{d}_l, r, \boldsymbol{\theta}') \; \mathrm{d}\mu_l = \nu_{lr}^2 + \rho_r^2, \tag{S.9}$$

we get

$$\begin{aligned} \mathbf{W}_r^* &= \frac{1}{p_r'} \sum_{l=1}^{L} p_{lr}' \left[\mathbf{d}_l \mathbf{d}_l' - \nu_{lr}(\mathbf{1}\mathbf{d}_l' + \mathbf{d}_l \mathbf{1}') + (\nu_{lr}^2 + \rho_r^2)\mathbf{1}\mathbf{1}' \right] \\ &= \rho_r^2 \mathbf{1}\mathbf{1}' + \frac{1}{p_r'} \sum_{l=1}^{L} p_{lr}' (\mathbf{d}_l - \nu_{lr}\mathbf{1})(\mathbf{d}_l - \nu_{lr}\mathbf{1})'. \end{aligned} \tag{S.10}$$

The relevant part for the optimization of the expected complete data log-likelihood (eq. S.6) with respect to μ_0, σ_0^2 is

$$Q_2(\mu_0, \sigma_0^2; \boldsymbol{\theta}') = -\frac{1}{2} \sum_{l=1}^{L} \sum_{r=1}^{R} p_{lr}' \left(\log \sigma_0^2 + \frac{1}{\sigma_0^2} \int_{-\infty}^{\infty} (\mu_l - \mu_0)^2 \; \pi(\mu_l|\mathbf{d}_l, r, \boldsymbol{\theta}') \; \mathrm{d}\mu_l \right).$$

Thanks to (eq. S.8) and (eq. S.9) we can explicitly solve

$$\frac{\partial}{\partial \mu_0} Q_2(\mu_0, \sigma_0^2; \boldsymbol{\theta}') = 0$$

and we obtain

$$\mu_0^* = \frac{1}{L} \sum_{l=1}^{L} \sum_{r=1}^{R} p_{lr}' \nu_{lr}. \tag{S.11}$$

Similarly, we solve

$$\frac{\partial}{\partial \sigma_0^2} Q_2(\mu_0^*, \sigma_0^2; \boldsymbol{\theta}') = 0$$

and get

$$\sigma_0^{2*} = \frac{1}{L} \sum_{l=1}^{L} \sum_{r=1}^{R} p_{lr}' \left(\rho_r^2 + (\nu_{lr} - \mu_0^*)^2 \right). \tag{S.12}$$

The new estimate for the mixing weights is

$$\pi_r^* = \frac{1}{L} \sum_{l=1}^{L} p_{lr}'. \tag{S.13}$$

2.1.2 Initial guess and choice of number of classes

We use the observed variance-covariance matrix of the transformed observed frequencies as an initial guess of the variance covariance matrix $\boldsymbol{W}_0$ and slightly perturb it to get initial guess for the other classes.

As it is unclear how many classes to use, we start with a rather large number of lasses (usually $R = 5$) and then reduce the number of classes until the proportion of loci $\pi_r > \pi^*$ is larger than a pre-defined threshold π^*. We found that a relative large value of $\pi^* = 0.2$ gave best results.

2.2 Step 2: Guessing initial branch length

The $\mathbf{W}_r$ inferred in the previous step are a function of two different components of our model, namely the variance-covariance given by the graph $\mathcal{G}$ through the branch lengths $\boldsymbol{c}$ and migration rates $\boldsymbol{w}$, as well as the variance that stems from binomial sampling of the data ($\boldsymbol{\Sigma}_l$). We therefore estimate the branch lengths $\boldsymbol{c}$ with the Nelder-Mead algorithm by minimizing the weighted Residuals Sums of Squares (RSS) between $\mathbf{W}_r$ and $\widehat{\boldsymbol{W}}_r = f(\boldsymbol{c})$ given by (7) of the main text, while accounting for $\boldsymbol{\Sigma}_l$. More precisely, let $\boldsymbol{c} = (c_1, \ldots, c_K)'$, define the vectorizations

$$\hat{w} = \mathrm{vect}(\widehat{\boldsymbol{W}}), \quad \boldsymbol{j}_k = \mathrm{vect}(\boldsymbol{J}_k),$$

and build the matrix

$$\boldsymbol{J} = (\boldsymbol{j}_1 | \dots | \boldsymbol{j}_K) \,.$$

We can, for each r, recast (7) into the form

$$\widehat{w} = \boldsymbol{J}\boldsymbol{c} + \bar{\boldsymbol{s}}. \tag{S.14}$$

with

$$\bar{\boldsymbol{s}} = \mathrm{vect}(\bar{\boldsymbol{\Sigma}})$$

where $\bar{\boldsymbol{s}}$ represents the vectorization of the weighted average of all $\boldsymbol{\Sigma}_l$ described in (10)

Before optimizing, the initial branch lengths c_k can be estimated with standard OLS from (eq. S.14). To do so, we need an initial $\widehat{\boldsymbol{W}}$ and $\boldsymbol{J}$. We calculate the observed variance covariance matrix and use it as the initial $\widehat{\boldsymbol{W}}$ for all r, similar to the initial $\boldsymbol{W}$. To build the initial $\boldsymbol{J}$, we randomly sample migration weights p from a uniform distribution or take values provided by the user. If any initial branch length is negative, it is set close to zero.

For optimization, we run Nelder-Mead to find the best combination of $\boldsymbol{c}$ and p that results in the minimum weighted RSS

$$\overline{RSS} = \sum_{r=1}^{R} \sum (\boldsymbol{W}_r - \widehat{\boldsymbol{W}}_r)^2 * \pi_r \tag{S.15}$$

The Nelder-Mead operates on $\log(c)$ and $\mathrm{logit}(p)$ space. If any proposed value is zero, a minimum value is reassigned before the log or logit transformation, similarly a maximum value other than one is reassigned before the logit transformation. To avoid issues with local minima, we run the Nelder-Mead a thousand times with different starting locations, and choose the branch length that result in the smallest RSS value.

3 References

Baum, L. E., Petrie, T., Soules, G., and Weiss, N. (1970). A Maximization Technique Occurring in the Statistical Analysis of Probabilistic Functions of Markov Chains. *The Annals of Mathematical Statistics*, 41(1):164–171.

Lange, K. (2010). *Numerical Analysis for Statisticians*. Statistics and Computing. Springer New York, New York, NY.

Murphy, K. P. (2012). *Machine learning: a probabilistic perspective*.

Nelder, J. A. and Mead, R. (1965). A simplex method for function minimization. *The Computer Journal*, 7(4):308–313.

Nocedal, J. and Wright, S. (2006). *Numerical Optimization*. Springer Science & Business Media.
